# High indirect bilirubin levels as an independent predictor of postoperative myasthenic crisis: a single-center, retrospective study

**DOI:** 10.3389/fneur.2023.1336823

**Published:** 2024-01-12

**Authors:** Chao Sun, Zhe Ruan, Yu Zhang, Rongjing Guo, Huanhuan Li, Tantan Wang, Ting Gao, Yonglan Tang, Na Song, Sijia Hao, Xiaoxi Huang, Shuang Li, Fan Ning, Yue Su, Qiang Lu, Qingqing Wang, Xiangqi Cao, Zhuyi Li, Ting Chang

**Affiliations:** ^1^Department of Neurology, Tangdu Hospital, The Fourth Military Medical University, Xi'an, Shaanxi, China; ^2^Department of Neurosurgery, Tangdu Hospital, The Fourth Military Medical University, Xi'an, Shaanxi, China; ^3^School of Pharmaceutical Sciences, Peking-Tsinghua Center for Life Sciences, Key Laboratory of Bioorganic Phosphorus Chemistry & Chemical Biology (Ministry of Education), Tsinghua University, Beijing, China; ^4^Department of Thoracic Surgery, Tangdu Hospital, The Fourth Military Medical University, Xi'an, Shaanxi, China

**Keywords:** postoperative myasthenic crisis, indirect bilirubin, biomarker, autoimmune, reactive oxygen species

## Abstract

**Background:**

Thymectomy is an efficient and standard treatment strategy for patients with myasthenia gravis (MG), postoperative myasthenic crisis (POMC) is the major complication related to thymectomy and has a strongly life-threatening effect. As a biomarker, whether the bilirubin level is a risk factor for MG progression remains unclear. This study aimed to investigate the association between the preoperative bilirubin level and postoperative myasthenic crisis (POMC).

**Methods:**

We analyzed 375 patients with MG who underwent thymectomy at Tangdu Hospital between January 2012 and September 2021. The primary outcome measurement was POMC. The association between POMC and bilirubin level was analyzed by restricted cubic spline (RCS). Indirect bilirubin (IBIL) was divided into two subgroups based on the normal upper limit of IBIL, 14 μmol/L.

**Results:**

Compared with non–POMC group, IBIL levels were significantly higher in patients with POMC. Elevated IBIL levels were closely associated with an increased risk of POMC (*p* for trend = 0.002). There was a dose-response curve relationship between IBIL levels and POMC incidence (*p* for non–linearity = 0.93). However, DBIL levels showed a U-shaped association with POMC incidence. High IBIL level (≥14 μmol/L) was an independent predictive factor for POMC [odds ratio = 3.47, 95% confidence interval (CI): 1.56–7.8, *p* = 0.002]. The addition of high IBIL levels improved the prediction model performance (net reclassification index = 0.186, 95% CI: 0.039–0.334; integrated discrimination improvement = 0.0345, 95% CI: 0.005–0.065).

**Conclusion:**

High preoperative IBIL levels, especially those exceeding the normal upper limit, could independently predict the incidence of POMC.

## Introduction

Myasthenia gravis (MG) is a typical autoimmune disease characterized by fluctuating muscle weakness. Over a 5-year period, thymectomy has significantly improved clinical outcomes and reduced prednisone requirements (1). Despite advances in medical treatment and surgical approaches, 3%−30% of patients with MG still experience postoperative myasthenic crisis (POMC) (2). POMC, the most severe complication of thymectomy, is characterized by a rapid worsening of symptoms, acute respiratory failure requiring mechanical ventilation, and even death. Several risk factors predicting POMC have been identified, including disease duration < 3 months, bulbar symptoms, vital capacity < 80%, preoperative myasthenic crisis (MC) history, and preoperative Osserman stages (IIb, III, and VI) (2, 3). In multiple autoimmunity diseases, the biomarkers from routine blood tests have been shown to be closely associated with the disease process (4–6). However, an easily accessible biomarker for POMC has not yet been proposed.

Bilirubin, generally considered the end product of heme metabolism, has potent anti-oxidative and anti-inflammatory effects (7). Indirect bilirubin (IBIL), also known as unconjugated bilirubin, originates mainly from the breakdown of red blood cells and is converted into direct bilirubin (DBIL) by UGT1A1 in the liver. Recent studies have shown that bilirubin could have opposite effects on human diseases, including coronary heart disease, cancer cachexia, and chronic obstructive pulmonary disease, depending on different bilirubin concentrations (8–10). Similarly, Zhang et al. (11) reported that low and high concentrations of bilirubin have opposite effects on nicotinic acetylcholine receptor (nAChR) function. However, the association between bilirubin levels and MG severity remains unclear. Yang et al. (12) revealed that low bilirubin levels may be a risk factor for MG aggravation, but Misra et al. (13) reported that high bilirubin levels are a biomarker of poor outcome in patients with MG. Bilirubin assays are routine, easily available preoperative examinations; therefore, bilirubin levels may have great clinical application as a biomarker for the prediction of POMC occurrence.

In this retrospective cohort study, we analyzed the dose-response association between preoperative bilirubin subtype levels and POMC incidence. In addition, we aimed to establish a threshold for bilirubin subtype levels to predict POMC occurrence.

## Methods

### Study design and participants

This was a single-center, retrospective study. We analyzed 474 patients with MG who underwent thymectomy at Tangdu Hospital between January 2012 and September 2021. The diagnosis of MG was based on fluctuating muscle weakness and fatigability, together with at least one of the following criteria fulfilled: (a) anti-AChR antibody positivity, (b) positive response to the neostigmine test, and (c) abnormal electrophysiological test results (repetitive nerve stimulation test and/or single-fiber electromyography). Thymic abnormalities were detected using chest computed tomography and magnetic resonance imaging of the thymic tissue. The exclusion criteria were as follows: (1) active infection or active or chronic inflammation within 4 weeks before the operation; (2) malignant tumor; (3) chronic hepatitis and cirrhosis; (4) alanine transaminase (ALT) and aspartate aminotransferase (AST) levels ≥2 times the upper limit of the normal ranges; (5) myasthenic crisis within 4 weeks before the operation; (6) thymoma recurrence; and (7) insufficient surgery information and missing IBIL, DBIL, and total bilirubin (TBIL) data.

This study was approved by the Ethics Committee of Tangdu Hospital, Fourth Military Medical University (reference number: 202111-16). The requirement for informed consent was waived for retrospectively collected data.

### Data collection

We retrospectively reviewed the following clinical data: sex, age at onset, age at thymectomy, preoperative MC history, disease duration, forced vital capacity (FVC) and vital capacity (VC) within 1 week before thymectomy, bulbar symptoms, and surgical approach (three-port, trans-sternal extended thymectomy, and video-assisted thoracic surgery). Bulbar symptoms were defined as difficulty swallowing and dysarthria. Three-port is a novel thymectomy approach created for thoracic surgery at Tangdu Hospital (14). MG severity was assessed using the Myasthenia Gravis Foundation of America (MGFA) classification within 1 week before surgery. Clinical data were extracted from patients' electronic medical records. Bilirubin data were collected within 1 week before surgery. Liver function tests were performed using venous blood samples after overnight fasting, and bilirubin levels were calculated in μmol/L. At our hospital, the normal ranges of TBIL, DBIL, and IBIL are 3.42–20.5, 0–6.84, and 0–14 μmol/L, respectively.

### Outcome measure

In this study, POMC was defined as the event of respiratory failure requiring intubation (>24 h) or re-intubation within 7 days after the operation. Respiratory failure is mainly caused by neuromuscular junction disorder (3). Other causes of respiratory failure, such as severely compromised cardiopulmonary systems, allergic diseases, and chest wall diseases, were excluded.

### Statistical analysis

The Kolmogorov–Smirnov test was used to assess the normality of the data distribution. Age at onset and thymectomy are expressed as the mean and standard deviation (SD). Disease duration, TBIL, DBIL, and IBIL levels are expressed as the mean (interquartile range). Categorical variables are reported as percentages or numbers. Continuous variables were compared between the groups using the independent *t*-test or Mann–Whitney *U*-test. Categorical variables were evaluated using the chi-square or Fisher exact test. All variables with a *p*-value < 0.1 detected in univariate logistic analyses or variables with important clinical significance were included in multivariate analyses. Odds ratios (ORs) were calculated using logistic regression analyses adjusted for MC history, lung function, MGFA classification, bulbar symptoms, surgical approach, and bilirubin subtype.

To test for linear trends across the quartiles, the median of bilirubin (TBIL, IBIL, and DBIL, respectively) was assigned to each quartile and modeled as a continuous variable. Furthermore, the association between bilirubin levels (TBIL, IBIL, and TBIL, respectively) and POMC incidence was evaluated using a restricted cubic spline (RCS) curve with four knots chosen at the 5th, 35th, 65th, and 95th quantiles based on logistic regression models. Based on the normal upper limit range (14 μmol/L), IBIL was divided into high and low IBIL subgroups. Since 22 FVC and VC values were not collected, 353 sample sizes were analyzed in binary logistic regression analysis.

Binary logistic regression analysis was adjusted to estimate the independent predictive effect of high IBIL levels on POMC. To evaluate the incremental value of the high IBIL subgroup for the prediction of POMC occurrence, the area under the curve (AUC), net reclassification index (NRI), and integrated discrimination improvement (IDI) values were calculated in the adjusted model.

Subgroup analyses were performed to determine the consistency of the association between high IBIL levels and POMC incidence across different subpopulations. Based on the likelihood ratio test, the *p*-values for the interaction terms were calculated by comparing the models with and without interaction terms. We did not conduct a subgroup analysis of MGFA classification I because of the small sample size of POMC and insufficient statistical power.

A two-tailed *p*-value < 0.05 was considered statistically significant. All analyses were performed using R statistical software (version 4.2).

## Results

### Study population

**Figure 1:**
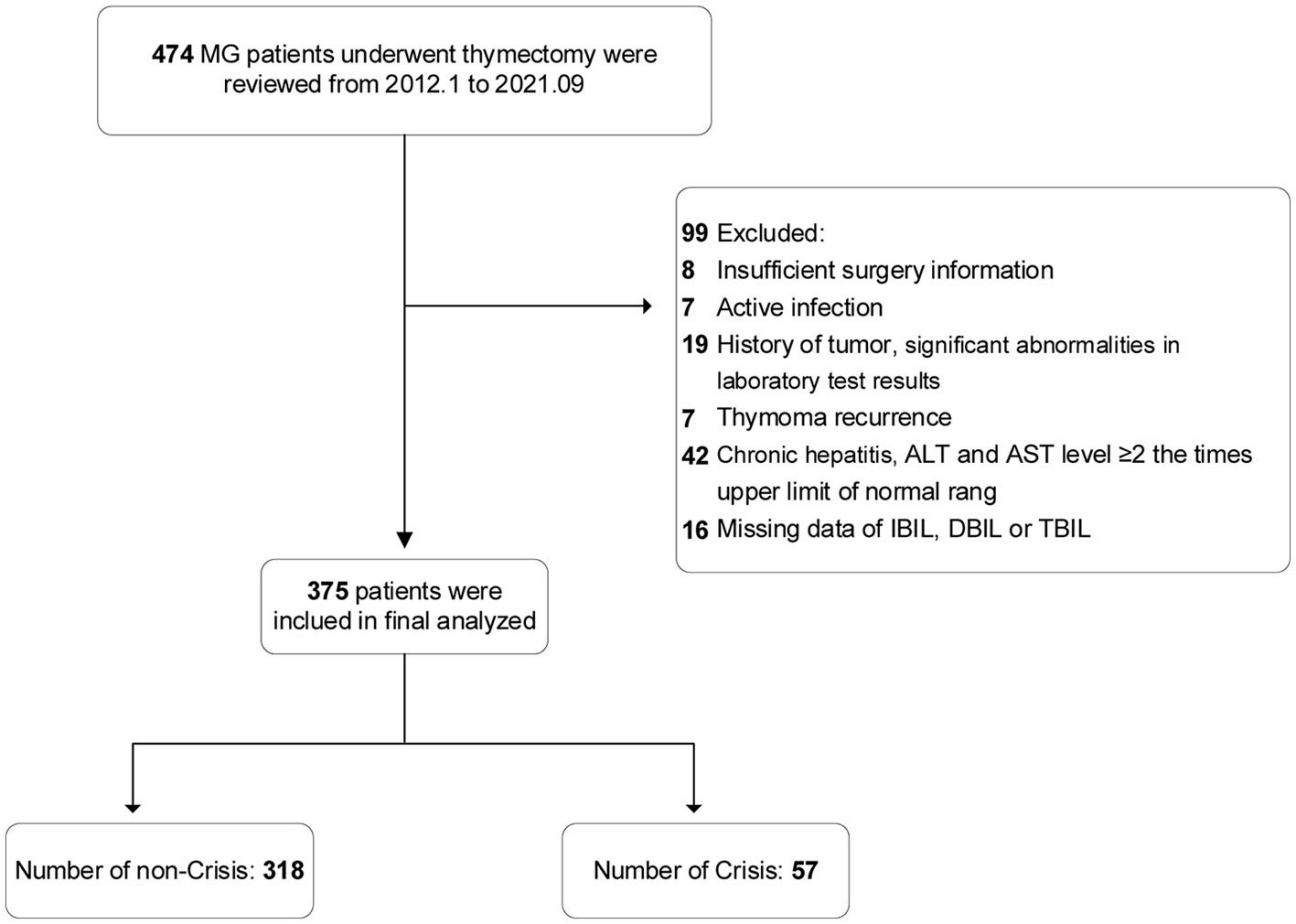
Flow diagram of participant inclusion and exclusion criteria.

In accordance with the predefined inclusion and exclusion criteria, 99 patients were excluded; the remaining 375 patients with MG who underwent thymectomy were finally included in the analysis (Figure 1). Of the eligible patients, the mean (SD) age at onset was 44.2 (14.7) years, mean (SD) age at thymectomy was 45.8 (14.7) years, 187 patients (49.9%) were female, and 57 patients (15.2%) developed POMC within 1 week.

Patients with POMC were more likely to have a history of MC, higher MGFA classification, bulbar symptoms, poor lung function, and experience a trans-sternal surgical approach. IBIL levels were significantly higher in patients with POMC. No significant differences were observed in age at onset, age at thymectomy, and sex between the Non-POMC and POMC groups. The other baseline characteristics of the patients are presented in Table 1.

**Table 1:** Baseline characteristics of all patients with MG.

	**Overal (*n* = 375)**	**Non-POMC (*n* = 318)**	**POMC (*n* = 57)**	***p*-value**
Age at onset, years, mean ± SD	44.2 ± 14.7	43.9 ± 14.9	45.8 ± 13.8	0.36
Age at thymectomy, years, mean ± SD	45.8 ± 14.7	45.5 ± 14.8	47.5 ± 14.2	0.35
Disease duration, months, median [IQR]	4 [1.0, 12.5]	4 [1.0, 12.0]	3 [1.0, 16.0]	0.75
**Disease duration**				
0–3 months *n* (%)	155 (41.3)	131 (41.2)	24 (42.1)	1
≥3 months *n* (%)	220 (58.7)	187 (58.8)	33 (57.9)	
**Sex**, ***n*** **(%)**				
Male	188 (50.1)	157 (49.4)	31 (54.4)	0.58
Female	187 (49.9)	161 (50.6)	26 (45.6)	
**MC history**, ***n*** **(%)**				
Yes	344 (91.7)	302 (95.0)	42 (73.7)	< 0.01
No	31 (8.3)	16 (5.0)	15 (26.3)	
**Lung function**, ***n*** **(%)**				
%VC or %FVC ≥80%	183 (48.8)	172 (54.1)	11 (19.3)	< 0.01
%VC or %FVC < 80%	170 (45.3)	130 (40.9)	40 (70.2)	
Missing	22 (5.9)	16 (5.0)	6 (10.5)	
**MGFA**, ***n*** **(%)**				
I	167 (44.5)	164 (51.6)	3 (5.3)	< 0.001
II	150 (40.0)	120 (37.7)	30 (52.6)	
III and IV	58 (15.5)	34 (10.7)	24 (42.1)	
**Bulbar symptoms**, ***n*** **(%)**				
Yes	281 (74.9)	249 (78.3)	32 (56.1)	0.001
No	94 (25.1)	69 (21.7)	25 (43.9)	
**Surgical approach**, ***n*** **(%)**				
Three ports	224 (59.7)	199 (62.6)	25 (43.9)	< 0.001
VTAS	63 (16.8)	57 (17.9)	6 (10.5)	
Trans-sternal	88 (23.5)	62 (19.5)	26 (45.6)	
Direct bilirubin μmol/L, median [IQR]	4.4 [3.2, 6.1]	4.4 [3.2, 6.0]	4.8 [2.7, 7.4]	0.63
Indirect bilirubin μmol/L, median [IQR]	9.3 [6.7, 13.1]	9.1 [6.7, 12.7]	10.2 [8.3, 16.7]	0.01
Total bilirubin μmol/L, median [IQR]	13.9 [10.8, 18.5]	13.5 [10.7, 17.8]	15.3 [11.5, 22.1]	0.03

MG, myasthenia gravis; POMC, postoperative myasthenic crisis; MC history, myasthenic crisis history; MGFA, Myasthenia Gravis Foundation of America classification; FVC, forced vital capacity; VC, vital capacity; VATS, video-assisted thoracic surgery; SD, standard deviation.

### Association of different bilirubin subtypes with POMC

To assess the relationship between different bilirubin subtype levels and POMC incidence, we applied quartiles to classify bilirubin levels into four categories. As shown in Table 2, increasing IBIL levels were correlated with the incidence of POMC. Compared with the lowest quartile of participants, the fully adjusted ORs for POMC incidence from the second to the fourth quartile in model 2 were 1.13 (0.34, 3.77), 2.91 (0.99, 9.19), and 4.43 (1.56, 13.82), respectively (*p* for trend = 0.002).

**Table 2:** Odd ratios (95% CI) for POMC incidence according to three types of serum bilirubin levels.

**Bilirubin types**	**Incidents/participants**	**Odds ratios (95% CI)**	
		**Model 1** ^a^	**Model 2** ^b^
**Indirect bilirubin (range; median**, μ**mol/L)**			
Quartile 1 (1.2–6.7; 5.41 μmol/L)	11/94	1.00 (Ref.)	1.00 (Ref.)
Quartile 2 (6.74–9.23; 8.07 μmol/L)	8/93	0.71 (0.26, 1.84)	1.13 (0.34, 3.77)
Quartile 3 (9.3–13.06; 10.6 μmol/L)	17/94	1.67 (0.74, 3.88)	2.91 (0.99, 9.19)
Quartile 4 (13.19–38.82; 16.72 μmol/L)	21/94	2.17 (0.99, 4.95)	4.43 (1.56, 13.82)
*p* for trend		0.012^*^	0.002^**^
**Direct bilirubin (range; median**, μ**mol/L)**			
Quartile 1 (0.02–3.12; 1.93 μmol/L)	17/89	1.00 (Ref.)	1.00 (Ref.)
Quartile 2 (3.2–4.39; 3.7 μmol/L)	7/95	0.34 (0.12, 0.83)	0.20 (0.06, 0.64)
Quartile 3 (4.4–6.04; 5.23 μmol/L)	15/94	0.81 (0.37, 1.73)	0.87 (0.31, 2.47)
Quartile 4 (6.1–17.39; 7.37 μmol/L)	18/97	0.97 (0.46, 2.03)	1.25 (0.5, 3.37)
*p* for trend		0.62	0.215
**Total bilirubin (range; median**, μ**mol/L)**			
Quartile 1 (3.8–10.72; 9.2 μmol/L)	12/94	1.00 (Ref.)	1.00 (Ref.)
Quartile 2 (10.8–13.9; 12.02 μmol/L)	8/93	0.63 (0.24, 1.64)	1.55 (0.49, 4.99)
Quartile 3 (13.91–18.42; 15.8 μmol/L)	13/94	1.10 (0.47, 2.58)	2.20 (0.72, 7.03)
Quartile 4 (18.49–43.78; 21.5 μmol/L)	24/94	2.34 (1.11, 5.17)	4.31 (1.61, 12.56)
*p* for trend		0.004^**^	0.003^**^

^a^Unadjusted model.

^b^Model 1 + adjusted for MC history, MGFA, Lung function, Bulbar symptom, Surgical approach.

^*^p < 0.05.

^**^p < 0.01.

Similar results were found for the association between TBIL levels and POMC incidence (*p* for trend = 0.003). Regarding DBIL, there was no increasing adjusted OR trend of POMC incidence from the second to the fourth quartile in model 2 (*p* for trend = 0.215), compared with the first quartile.

### Association between high IBIL levels and POMC

Multiple clinical studies have shown that high IBIL levels negatively affect human health, but the threshold concentration remains uncertain (8, 10, 13, 15). In an *in vitro* study, Bianco et al. (16) reported that when the IBIL level exceeded 15 μmol/L, it caused cytotoxicity in H5V and HK2 cells. Univariable and multivariable linear regression model were used to assess the relationship between different bilirubin subtype levels and POMC incidence.

**Figure 2:**
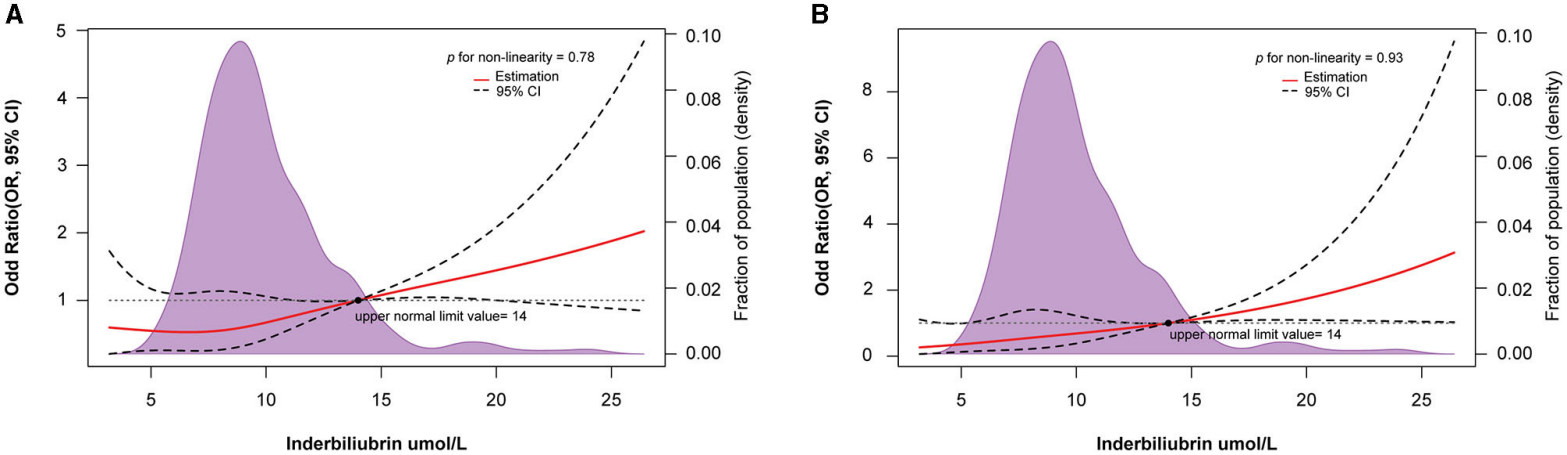
Restricted spline curve of the IBIL level and odd ratio of POMC in MG patients: **(A)** Unadjusted model; **(B)** Adjusted model, adjusted for MC history, MGFA, lung function, Bulbar symptoms, and surgical approach.

According to the results of the RCS curve, a dose-response relationship was observed between the IBIL levels and POMC incidence (*p* for nonlinearity = 0.78; Figure 2A). At the same time, a linear association existed in the multivariable model adjusting MC history, MGFA classification, lung function, bulbar symptoms, and surgical approach (*p* for nonlinearity = 0.93; Figure 2B). In this study, 14 μmol/L was used as the upper normal limit. Interestingly, the part exceeding 14 μmol/L had a stronger line dose-response for POMC incidence in the unadjusted and adjusted model.

TBIL levels were linearly associated with a higher risk of POMC in the adjusted model (*p* for nonlinearity = 0.813; Supplementary Figure S1D). However, a U-shaped relationship between TBIL levels and POMC occurrence was found in the unadjusted model (Supplementary Figure S1C). The exposure-response relationship between DBIL levels and the risk of POMC incidence was U-shaped in the unadjusted and adjusted models (*p* for nonlinearity = 0.002 and *p* for nonlinearity = 0.037, respectively; Supplementary Figures S1A, B). Interestingly, when the TBIL and DBIL levels exceeded the normal upper limit (6.84 and 20.5 μmol/L, respectively), the risk of POMC increased.

### Predictive role of high IBIL

**Figure 3:**
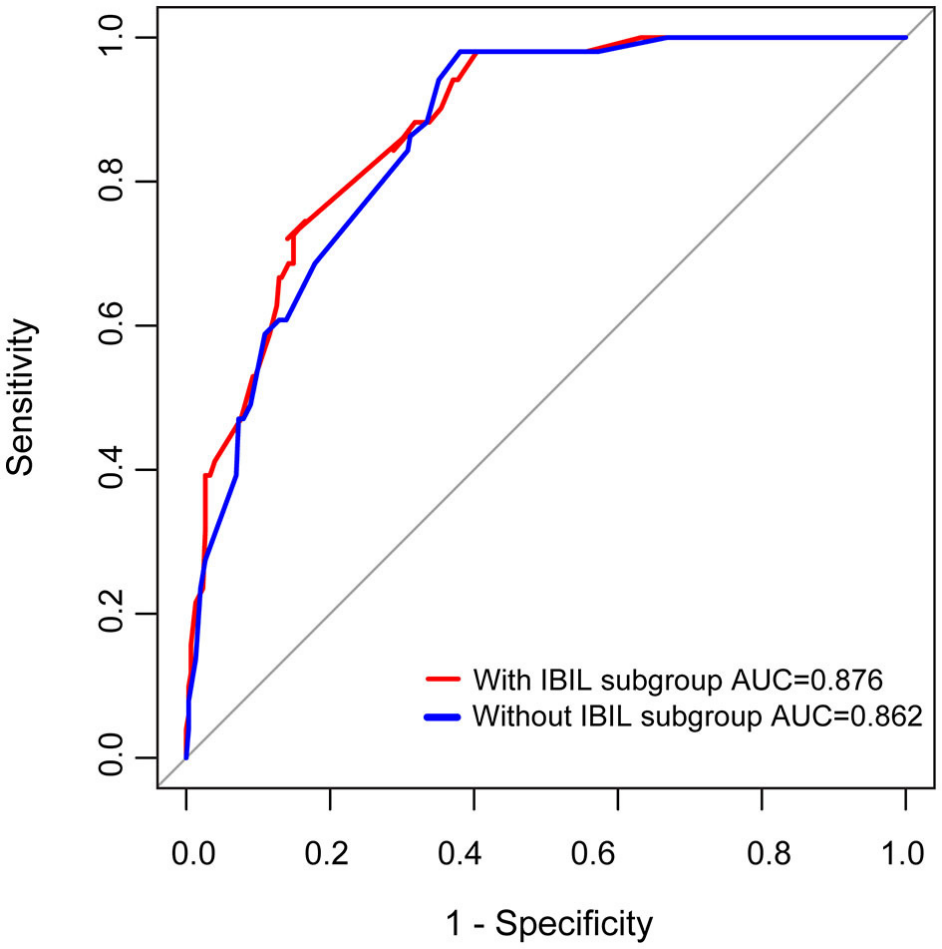
ROC curves of prediction for POMC. MC history, MGFA, Lung function, surgical approach, and IBIL subgroup as variables were analyzed by the binary regression model.

Previous studies have found that preoperative history of MC, preoperative bulbar symptoms, MG Osserman stage, lung function, thoracotomy, disease duration, and other clinical characteristics were independent risk factors for POMC. Furthermore, Kanai et al. (3) developed a clinical predictive score for POMC based on clinical characteristics. In this study, we firstly found that as the biomarker, a high IBIL level was also an independently predictive biomarker of POMC (OR = 3.47, 95% CI: 1.56–7.8; *p* = 0.002; Table 3). The predictive power of the basic clinical characteristics model for the IBIL subgroup was measured using receiver operating characteristic curves, and the AUC value was 0.876. When the IBIL high group was removed from the basic model, the AUC value decreased to 0.862 (Figure 3). Moreover, the addition of a high IBIL index had a significant incremental value for predicting POMC in terms of the NRI (0.186, 95% CI: 0.039–0.334) and IDI (0.0345, 95% CI: 0.005–0.065; Table 4).

**Table 3:** Multivariate analyses of association between risk factors and POMC by binary logistic regression.

**Risk factors**	**OR**	**95% CI**	***p-*value**
MC history	4.11	1.5–11.5	0.006
%VC or %FVC < 80%	4.15	1.88–9.91	< 0.001^**^
**MGFA**			
I	1 (Ref.)		
II	9.46	2.95–42.5	< 0.001^***^
III&IV	32.5	8.55–165	< 0.001^***^
Bulbar symptoms	0.53	0.23–1.18	0.13
**Surgical approach**			
Three ports	1 (Ref.)		
VTAS	0.71	0.2–2.14	0.6
Trans-sternal	3.3	1.49–7.5	0.004^**^
High IBIL	3.47	1.56–7.8	0.002^**^

^**^p < 0.01.

^***^p < 0.001.

**Table 4:** Incremental predictive value of high IBIL for POMC incidence.

	**Discrimination**		**Reclassification**			
	**C-statistic (95% CI)**	***p-*** **value**	**NRI (95% CI)**	***p-*** **value**	**IDI (95% CI)**	***p-*** **value**
Without IBIL^a^	0.862 (0.818–0.907)	**–**	1.00 (Ref.)	**–**	1.00 (Ref.)	**–**
With IBIL^b^	0.876 (0.833–0.919)	0.169	0.186 (0.039–0.334)	0.014^*^	0.0345 (0.005–0.065)	0.024^*^

^a^Without IBIL model: MC history, MGFA, lung function, surgical approach.

^b^With IBIL model: MC history, MGFA, lung function, surgical approach, and IBIL subgroup.

^*^p < 0.05.

A nonlinear correlation was observed between DBIL levels and POMC incidence; therefore, the DBIL subgroup was not included in the prediction model of POMC. Additionally, the TBIL subgroup was not analyzed in the prediction model of POMC because the higher TBIL group was mainly attributed to the increased IBIL level (Supplementary Figures S2A, B).

### Stratified analyses of the association between high IBIL and the risk of POMC incidence

**Figure 4:**
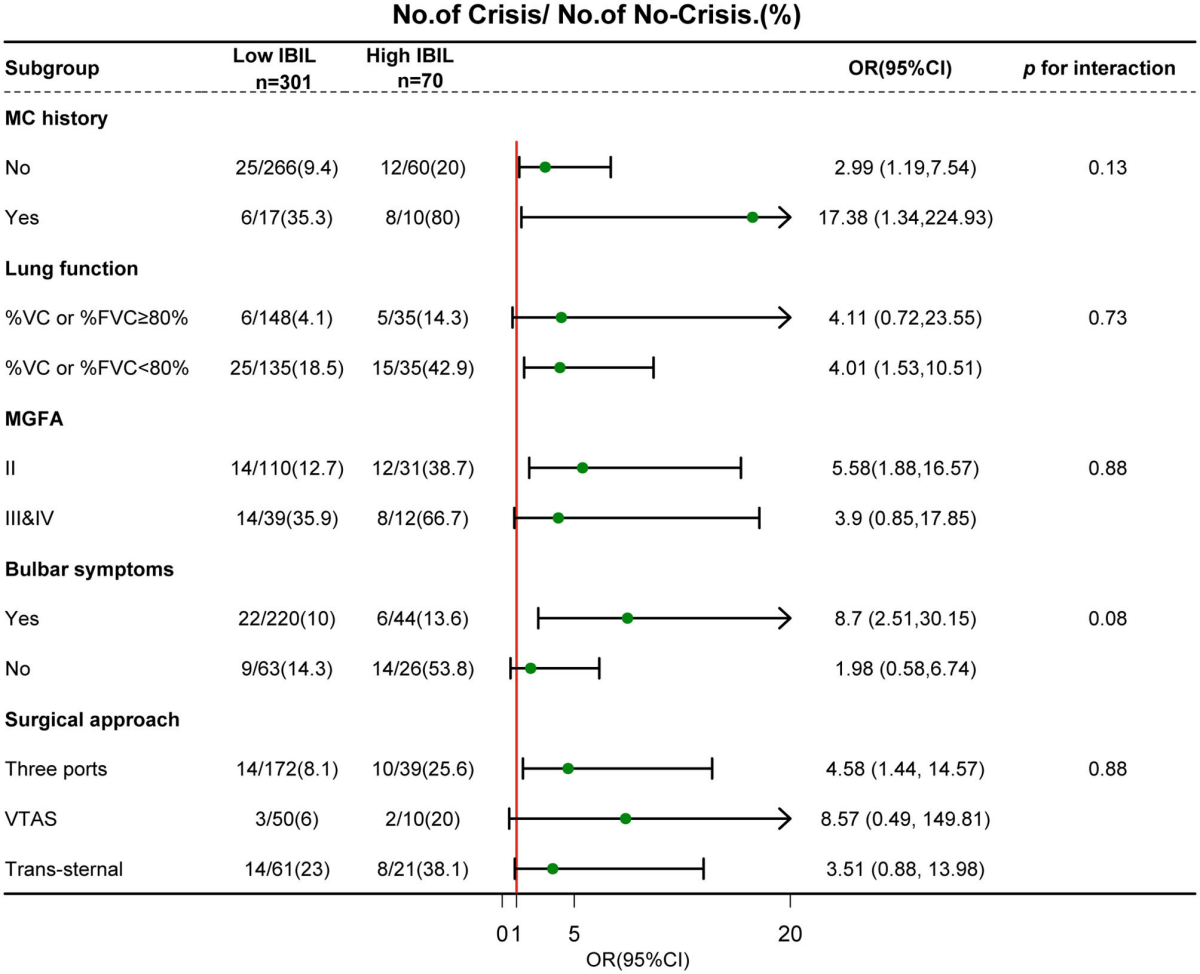
Subgroup analyses of the association between IBIL and POMC according to risk factors. Interactions between IBIL and interesting factors on the POMC were tested by the likelihood ratio test in the adjustment regression model. A forest plot depicts OR and 95% CI of POMC incidence in different subgroups.

To determine significant interactions for the association between IBIL and POMC incidence, we stratified the individuals according to MC history, lung function, bulbar symptoms, surgical approach, and MGFA classification (Figure 4). Subgroup analysis showed that a weak dose-response relationship between IBIL and the risk of POMC incidence was more pronounced in MC history (*p* = 0.13) and bulbar symptoms (*p* = 0.08). This finding was consistent with the fact that MG patients with poor outcomes have higher IBIL levels (13).

## Discussion

This is the first study to investigate the association between preoperative individual bilirubin subtype levels and POMC incidence. Preoperative IBIL levels were higher in the POMC group than in the Non-POMC group, and a linear dose-response relationship was shown between the IBIL levels and POMC incidence. Interestingly, the IBIL level, especially that exceeding the normal upper limit, was an independent risk factor for POMC progression after controlling for the classic clinical risk factors. Therefore, high preoperative IBIL levels may serve as a routinely available biomarker for POMC incidence.

POMC is the most severe complication of thymectomy, and patients need mechanical ventilation and intensive care unit care. Thus, the early identification of risk factors is critical for reducing POMC occurrence. Lung function, bulbar symptoms, MGFA classification, and disease duration are classic clinical risk factors, but the proportion of patients with a disease duration < 3 months was not significantly different between the Non-POMC and POMC groups in our study. Moreover, a previous report showed that a history of MC was also a risk factor for POMC (2), which is also consistent with our findings. Although the RR-AChR antibody, miR-150-5p, and complement levels have emerged as potential biomarkers to predict MG progression (17, 18), convenient and inexpensive biomarkers are urgently needed.

Bilirubin was previously believed to have potent antioxidant and protective effects against autoimmune diseases. However, the correlation between bilirubin levels and MG severity remains inconsistent. Yang et al. (12) showed that serum IBIL and DBIL levels were lower in patients with MG than in healthy controls. IBIL and DBIL levels were correlated with disease severity and MGFA classification. Misra et al. found that serum bilirubin levels were increased in MG patients with poor outcomes (MGFA classifications III, IV, and crisis). Although this retrospective study had a longer follow-up period (1991–2016), only 81 patients' data were included (13). In our study, preoperative IBIL levels were significantly higher in the POMC group than in the Non-POMC group. In RCS regression analysis, line-response relationships were found in both TBIL and IBIL with POMC after adjusting for clinical variables. However, a U-shaped relationship was observed between DBIL and POMC in the adjusted model. Because IBIL accounts for the majority of TBIL, the relationship between TBIL and POMC incidence may be attributed to increased IBIL levels. Compared with the first quartile, the second quartile of IBIL level was negatively correlated with POMC incidence in the unadjusted model (Figure 2A). Hence, the risk threshold of the IBIL level is important in POMC progression for clinical judgment.

Recently, bilirubin has been found to have both anti-oxidant and pro-oxidant effects at different concentrations. Mild hyperbilirubinemia could protect against cellular reactive oxygen species (ROS) damage and have a beneficial effect in autoimmune diseases, including systemic lupus erythematosus, diabetes, and inflammatory bowel disease (IBD) (6, 19, 20). However, severely high bilirubin levels can cause permanent neurological impairments in neonates. Peng et al. found that DBIL dose-dependently predicted poor outcomes in patients with acute ischemic stroke after thrombolysis (21). When the total bilirubin level exceeded 13 μmol/L, the risk of COPD and CHD increases (8, 10). Hence, the beneficial or harmful effect on normal physical function or disease may depend on the IBIL concentration, but there is no certain threshold.

*In vitro* experiments, when the IBIL level exceeds 3.6 μmol/L, especially 15 μmol/L, Bianco et al. (16) reported that IBIL has significant pro-oxidant and cytotoxic effects. In our study, all IBIL data were obtained from our hospital, and the normal range for IBIL was 0–14 μmol/L. The starting range of severe cytotoxic effects of IBIL is approximately equal to the upper limit of its normal range, indicating that exceeding the upper limit of IBIL is harmful to immune homeostasis. Interestingly, high IBIL levels (>14 μM) were significantly related to an increased incidence rate of POMC. Compared with the Youden index, the upper limit value of the IBIL level was easily obtained from clinical data and had more clinical judgment value for POMC prediction. Therefore, with 14 μmol/L as the cut-off value, the patients with MG were divided into high and low IBIL subgroups. By controlling for clinical risk factors, a high IBIL level was an independent predictive biomarker for POMC. In patients with acute ischemic stroke after thrombolysis, increased DBIL before thrombolysis has a greater incremental predictive value for poor outcomes (21). Similarly, based on the IDI and NRI analyses, we found that predictive ability can be significantly improved by including the IBIL variable in the model.

IBIL is covered by DBIL in the liver. MG is also a systemic autoimmune inflammatory disease that can accompany non-motor symptoms, including headaches, sleep disturbances, and even taste disorders (22). In this study, patients with chronic hepatitis and abnormal ALT or AST levels were excluded. Although liver injury was reported in MG, few liver functions were observed in patients with non-motor symptoms. Immune-erythroid cells collected from bone marrow can promote the CD11b^+^ human peripheral blood mononuclear cells to produce proinflammatory cytokines (23). Shi et al. (24) found that the bone marrow hematopoietic stem could increase the number of monocytes and neutrophils, exacerbating central nervous system inflammatory injury in multiple sclerosis. These findings indicate that red blood cells may have immunomodulatory roles in autoimmune diseases, including MG. Hence, we could reasonably speculate that the imbalance of immunity of MG influences red blood cell and liver function, leading to elevated IBIL levels.

High IBIL levels are associated with a higher risk of POMC, and IBIL levels could promote MG progression via direct or indirect mechanisms. Zhang et al. found that IBIL can directly affect AChR function in superior cervical ganglion. Low IBIL concentrations could increase the peak amplitude of nicotinic AChR channel currents, but high concentrations of IBIL (3, 4, or 5 mM) could inhibit the function of AChR (11). The AChR subtype in the superior cervical ganglion is different from that of the neuromuscular junction. Whether high concentrations of IBIL directly affect AChR function needs to be explored in electrophysiological assays. MG is a typical T cell-dependent, B cell-mediated autoimmune disease. High IBIL levels can induce cells to produce excess ROS, which is considered an important bridge between innate and adaptive immunity (25). Mitochondrial ROS contribute to the maintenance of CD4 and CD8 T cells. Increased mitochondrial O_2_^**.−**^ in T cells show higher levels of interleukin (IL)-4, IL-16, IL-17, tumor necrosis factor α, and inferno γ (26). Experimental autoimmune MG rats with diabetes experienced more severe clinical symptoms and increased Tfh, Th17, and memory B cell populations (27). This may be because high glucose intake induces the formation of mitochondrial-derived ROS, leading to the differentiation of Th17 cells through tumor growth factor-β activation (28). Additionally, ROS levels are much higher in activated B cells, which is essential for B cell proliferation (29). Gilljam et al. (30) revealed that ROS could play an important role in sustaining high immunoglobulin production when TLR9 induces B activation. ROS, as secondary messengers, can directly or indirectly act on various transcription factors, such as PKC/NF-κB, PI3K/AKT/mTOR, and RAS/ERK/AP-1 signaling pathways to mediate B cell and T cell differentiation (29, 31). The aforementioned findings suggest that ROS could induce T cell and B cell activation and promote autoimmune disease progression. Immune cell metabolism is central to inducing B and T cell differences; however, the characterization of metabolism in MG is still unknown. This study showed that high IBIL levels aggravate MG symptoms, which may depend mainly on the production of large amounts of ROS.

Recently, bilirubin nanoparticles have shown therapeutic efficacy in cardiac ischemia or reperfusion injury, rheumatoid arthritis, and IBD mouse models (32–34). These effects are mainly due to potent ROS scavenging, endogenous antioxidant molecules, and anti-inflammatory properties (35). Herein, when the IBIL level exceeded the normal upper limit, we found that the adverse effect of bilirubin was apparent in the progression of MG. Therefore, it is reasonable to speculate that bilirubin nanoparticles may not reach therapeutic efficacy when bilirubin levels are abnormally high in the body. Furthermore, bilirubin nanoparticles may cause ROS production and inflammation when an excess dose of bilirubin nanoparticles is administered.

In this study, exceeding the normal limits of IBIL and DBIL significantly increased the risk of POMC. However, the observed dose-response relationship was solely evident in the unadjusted and adjusted models of unconjugated, lipid-soluble bilirubin (IBIL), potentially due to its lipid-solubility and ability to elicit a robust level of reactive oxygen species (ROS) at low concentrations. Unlike water-soluble direct bilirubin (DBIL), IBIL can permeate any cell by virtue of its ability to readily diffuse through the phospholipid bilayer, thereby influencing mitochondrial reactions, augmenting ROS levels, and even provoking autoreactive immune cell activation.

Our study has several limitations. First, this was a retrospective, single-center study; therefore, different zones and ethnic groups should be considered. Second, although multiple reported POMC risk factors were collected, the reason for the increased IBIL levels in patients with POMC remained unclear. Abnormal liver function and some erythroid cell subsets with immunomodulatory functions should be analyzed during MG progression. Third, the correlation between high IBIL levels, ROS levels, and immune cell activation should be explored in an *in vitro* experiment, and further *in vivo* experiments are needed to confirm this mechanism. Finally, this study only concerned MG status during inpatient care, so a longer observation period would be useful to evaluate the association between high IBIL levels and MG progression. The IBIL level in myasthenic crisis that is not caused by thymectomy should be further confirmed.

## Conclusion

We found that high preoperative IBIL levels, especially those exceeding the normal range (≥14 μmol/L), are an easily accessible and cost-effective biomarker for predicting POMC incidence. The relationship between IBIL levels and MG progression should be investigated over a longer follow-up period and through *in vitro* functional experiments.

## Data availability statement

The raw data supporting the conclusions of this article will be made available by the authors, without undue reservation.

## Ethics statement

The studies involving humans were approved by the Ethics Committee of Tangdu Hospital, Fourth Military Medical University (reference number: 202111-16). The studies were conducted in accordance with the local legislation and institutional requirements. The Ethics Committee/institutional review board waived the requirement of written informed consent for participation from the participants or the participants' legal guardians/next of kin.

## Author contributions

CS: Conceptualization, Data curation, Funding acquisition, Investigation, Supervision, Writing—original draft, Writing—review & editing. ZR: Methodology, Supervision, Writing—original draft. YZ: Supervision, Writing—original draft. RG: Data curation, Investigation, Writing—original draft. HL: Data curation, Investigation, Writing—original draft. TW: Data curation, Methodology, Writing—original draft. TG: Data curation, Investigation, Writing—original draft. YT: Data curation, Investigation, Writing—original draft. NS: Formal analysis, Writing—original draft. SH: Data curation, Project administration, Writing—original draft. XH: Investigation, Project administration, Writing—original draft. SL: Data curation, Project administration, Writing—original draft. FN: Data curation, Project administration, Writing—original draft. YS: Data curation, Project administration, Writing—original draft. QL: Supervision, Writing—original draft. QW: Investigation, Project administration, Writing—original draft. XC: Investigation, Project administration, Writing—original draft. ZL: Supervision, Writing—original draft, Writing—review & editing. TC: Project administration, Supervision, Writing—review & editing, Writing—original draft.
