# Supplementary material for: High indirect bilirubin levels as an independent predictor of postoperative myasthenic crisis: a single-center, retrospective study
Source: Front Neurol. 2024 Jan 12;14:1336823. doi: 10.3389/fneur.2023.1336823 (PMC10811789; doi:10.3389/fneur.2023.1336823)
Supplement: Supplementary file 1 [file Data_Sheet_1.docx]

**Supplementary materials**


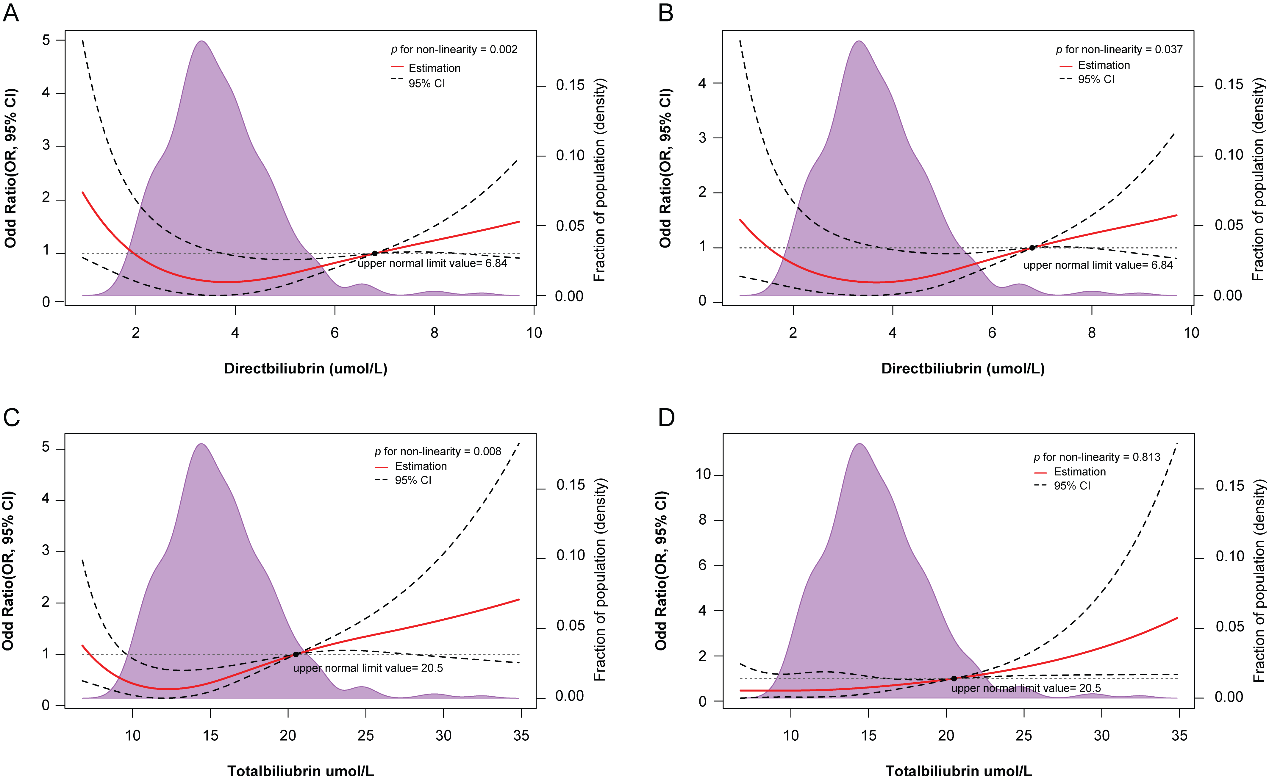


**Fig. S1** Restricted cubic spline curve for association between DBIL, TBIL and the risk of POMC. (A) unadjusted model of DBIL; (B) adjusted model of DBIL; (C) unadjusted model of TBIL; (D) adjusted model of TBIL. Adjustments were made for MC history, MGFA, Lung function, Bulbar symptoms, Surgical approach.


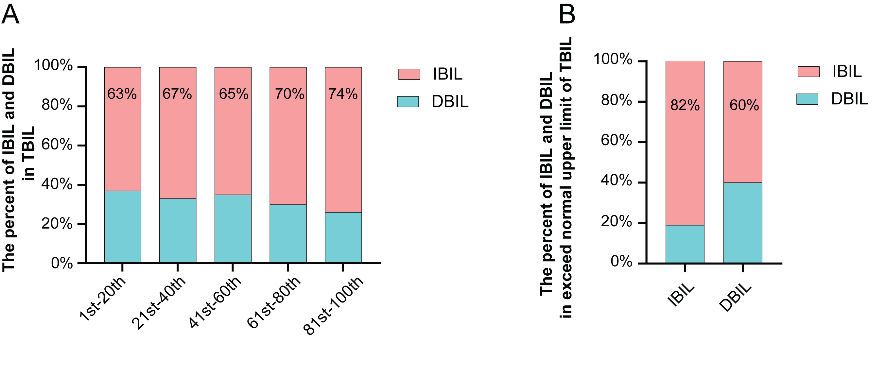


**Fig. S2** The proportion of IBIL and DBIL in TBIL. (A) TBIL was categorized into five centiles; (B) Exceed the upper limit of normal (TBIL, 20.5 μmol/L).
